# Supplementary material for: Lyso-Gb3 Increases αvβ3 Integrin Gene Expression in Cultured Human Podocytes in Fabry Nephropathy
Source: J Clin Med. 2020 Nov 13;9(11):3659. doi: 10.3390/jcm9113659 (PMC7696179; doi:10.3390/jcm9113659)
Supplement: Supplementary file 1 [file jcm-09-03659-s001.pdf]

Journal of
Clinical Medicine

Supplementary files

Lyso-Gb3 Increases αvβ3 Integrin Gene Expression in Cultured Human Podocytes in Fabry Nephropathy

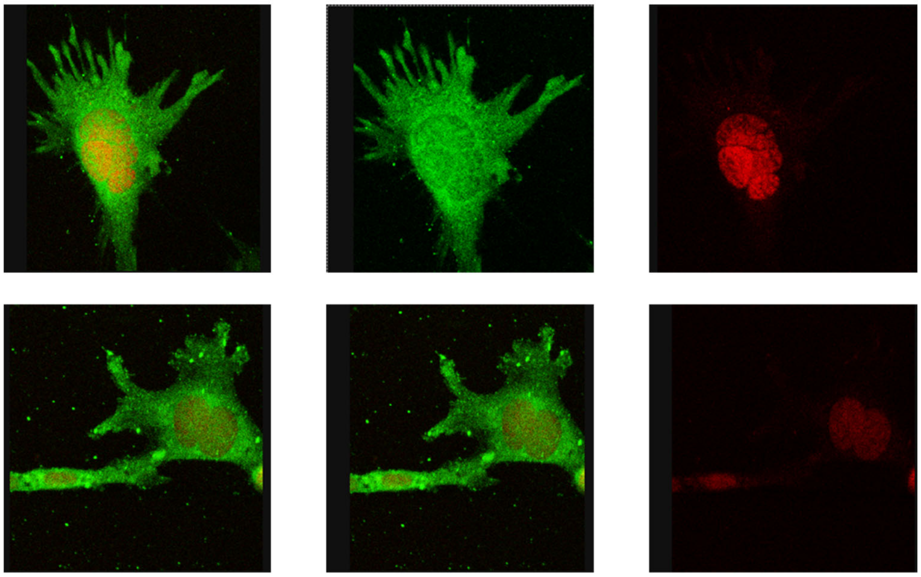

Figure S1. Densin. Morphology of cultured human podocytes and expression of the slit diaphragm protein densin using a previously described antibody (Ahola H, Heikkliä, Aström E, et al. A novel protein, densin, expressed by glomerular podocytes. J Am Soc Nephrol. 2003; 14(7):1731–1737.

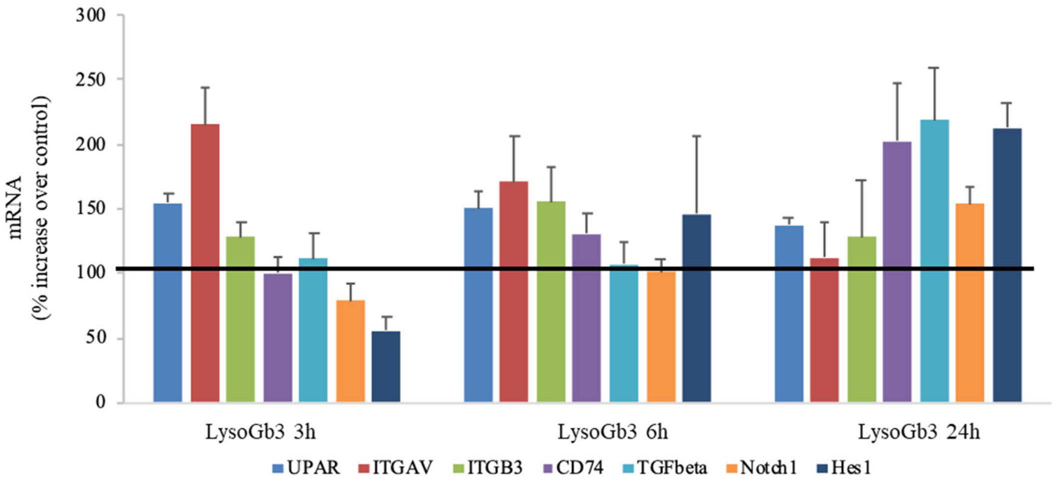

.

Figure S2. Time course of gene expression in podocytes in response to lyso-Gb3. The Figure present data shown in Figure 3, with SEM bars added.
